# Supplementary material for: A Protocol for a Mixed-Methods Process Evaluation of a Local Population Health Management System to Reduce Inequities in COVID-19 Vaccination Uptake
Source: Int J Environ Res Public Health. 2022 Apr 11;19(8):4588. doi: 10.3390/ijerph19084588 (PMC9029748; doi:10.3390/ijerph19084588)
Supplement: Supplementary file 1 [file ijerph-19-04588-s001.zip › ijerph-1638862-SI.pdf]

Supplementary file:
Interview guide example

How is NCL's Population Health Management platform *HealtheIntent* being used achieve its stated aims?

Interview Schedule

Study: *How is NCL's Population Health Management platform HealtheIntent being used achieve its stated aims?*

<u>Introduction</u>

Thank you for agreeing to participate in this interview. We are conducting this research to capture what health and care professionals in NCL think about the HealtheIntent COVID-19 Vaccination Dashboard. This dashboard will be used as a case study in the wider evaluation of HealtheIntent. We are interested in your experiences and opinions and there are no right or wrong answers. While our questions here are on the Covid dashboard please do feel free to draw upon or compare with your experience of other HealtheIntent tools or others similar tools that you have used.

Participation in this interview is entirely voluntary and there will be no consequences if you choose to withdraw. All your responses will be confidential. They will be de-identified, securely stored and they will not be shared. You can decline to answer any question or stop the interview at any time and for any reason.

As outlined in the information sheet, what you tell us will be anonymised so you cannot be identified in any analysis/reports that are written and your participation in the study will remain confidential. However, if you indicate that you or someone else is at risk of harm, the researcher has a duty to report this to relevant agencies. The researcher will discuss this with you first, wherever possible.

If you say anything that you would prefer would be kept off the anonymised record, please let us know. If you say something that I think might be sensitive, I will check with you whether you want this excluded from analysis. Such comments often provide useful context so there may be benefit in sharing them off the anonymised record.

The interview should take approximately 45 minutes to an hour depending on how much you would like to share. If you consent, I would like to record this interview today – may I?

Do you have any questions?

<u>Demographics</u>

First, I would like to ask you two quick demographic questions please. This information will be anonymised.

1. Can you please tell me what kind of access you have to the Covid vaccination dashboard?

   Prompt: Access to identifiable patient data, access to aggregate statistics, no direct access but recipient of data via a third party

2. Can you please confirm your job title?

Supplementary file:
Interview guide example

Prompt: Provider (general practice, social care, mental health trust, acute trust, community trust); Planner (Public Health, PCN); Commissioner (CCG)

Consent

Please can you confirm that you have signed, returned and been provided with a signed copy of the informed consent sheet?

Thank you for your consent. Please do remember that your consent can be withdrawn at any time during this interview, but shortly after the interview is finished your data will be anonymised. This will make it impossible to remove your data.

The interview today is structured into four sections: first I'll ask about your personal usage of the tool, then we'll discuss its design, then we'll talk about the impact of the tool, and finally we'll talk about comparisons to other tools and providers. We might move away from this structure depending on what makes sense to you.

Supplementary file:
Interview guide example

Motivation & individual usage

1. What motivated you to use the dashboard?
    a. purpose of using it (managerial, strategic, analytical, or commissioning)
    b. ease of access, usability, relevant data, timely data
    c. System factors: leaders, champions, training

2. What inhibits/stops you using it?
    a. Prompts: ease of access, usability, relevant data, timely data; trusting the data; IT infrastructure; competing priorities; opinions of others

3. How did you first hear about the dashboard?
    a. Prompts: comms channels; meetings; colleague recommendation; part of HealtheIntent team

4. How consistently have you used the dashboard?
    a. *NB: if interviewee has different roles, explore both – e.g. a GP in clinical practice vs. strategic commissioner in CCG*

    b. Prompts: over time, for similar purposes, in similar ways
    c. Prompts: What factors led to your change in use of the dashboard

5. [Low priority] How did you find integrating the dashboard into your usual day-to-day work?
    a. Prompts: new habit, attached to existing habits, prompted by external factors e.g. meetings/reporting (how automatic is it for the dashboard user to turn to the tool)
    b. Prompts: facilitators and barriers

Design of the tool

6. Tell me how you have found using the tool.
    a. Prompts: How well do the different pages of the COVID vaccination dashboard work do what you want them to?
        (i) the overview page (current uptake information for different care teams and patient groups)
        (ii) the demographics and equalities page (to identify inequity)
            1. Viewing demographic data over time
        (iii) the data quality element
        (iv) other pages

7. Which elements of the tool do you find the most and least helpful?
    a. *NB: elements might be pages, filters, role-based access, comparability to other data sources, ability to extract data/tables etc.*

    b. Prompts: Most/least helpful for your purpose (managerial, strategic, analytical, or commissioning)

8. The dashboard has been changed several times, for example to include filters for bedbound or housebound people. Have you been aware of those changes and what do you think of them?

   a. Prompt: How were you made aware of the changes?
   b. Prompt: Were changes for the better or worse?

9. Are there any ways you think the COVID vaccination dashboard could be improved?
   a. Prompts: format, usability, interoperability, accessibility, etc.

Impact of tool

10. Has using the dashboard changed the way you do things? Or the way things are done around here?
    a. Prompt: If yes, what do you do (or what is done) differently since having access to data through the COVID vaccination dashboard?
       - How does it affect
         (i) your understanding of the local population?
         (ii) your ability to identify opportunities for improvement?
         (iii) your strategic decisions and planning?
         (iv) the way you work with others?
         (v) your workload?

    b. Prompts: If no, what are the reasons for this?
         (i) The dashboard tool itself
         (ii) National policy
         (iii) Local policy
         (iv) Other data
         (v) Lack of usage

11. What, if anything, do you do differently to identify and respond to inequity since having access to data through the COVID vaccination dashboard?
    a. *NB: there is a demographic and inequalities page allowing comparison of e.g. uptake by demographic factors*
    b. *NB: actions could be of individual user or user's organisation/team (e.g. a GP speaking to patients or involved in practice-level outreach)*

    c. Prompts: If yes, please tell me more about these changes/actions taken
    d. Prompts; If no, could you tell me reasons it has not impacted on your work/decisions in this way?

Comparisons to other systems/providers

12. Information on vaccine uptake is available in other ways in NCL. How do you use the COVID vaccination dashboard compared to other platforms, like Foundry?
    a. Prompts: What function does each platform serve for the user regarding COVID vaccination?
    b. Prompts: How useful is the COVID vaccination dashboard compared to other platforms?
    c. Prompts: Which tool is preferred and why?
    d. Prompts: Do you use the tools in combination and if so how?

14. The COVID vaccination dashboard provides a system view in two ways: (1) aggregate data about the whole NCL population, and (2) data from multiple providers on each individual. To what extent do you find having these system views helpful?
    a. Prompts: How does the system-level view affect how you use the dashboard?
    b. Prompts: How does the ability to benchmark/compare influence how you use the dashboard?
    c. Prompts: How has having system-wide information influenced how you work with other organisations?
    d. Prompts: How does access to this information affect how you plan/make decisions?

Close

15. **To sum up, what is your perception of the overall effect of the COVID vaccination dashboard on direct care and system working?**

16. Is there anything else you'd like to share with me?

Thank you very much for your time and the information you have shared with me today.

We are still recruiting interview participants and would be interested in any recommendations you have for colleagues we might invite?

Finally, if we have any questions when reviewing the interview transcript would you be amenable to us following up with you at a later point?
